# Conserved contributions of NMDA receptor subtypes to synaptic responses in lamina II spinal neurons across early postnatal development

**DOI:** 10.1186/s13041-020-00566-9

**Published:** 2020-03-05

**Authors:** Hadir Mahmoud, Newton Martin, Michael E. Hildebrand

**Affiliations:** 1 Department of Neuroscience, Carleton University, Ottawa, ON Canada; 2 Neuroscience Program, Ottawa Hospital Research Institute, Ottawa, ON Canada; 3 Brain and Mind Research Institute, University of Ottawa, Ottawa, ON Canada

**Keywords:** Spinal cord, Dorsal horn, Lamina II, Synapse, NMDA receptor, GluN2A, GluN2B, GluN2D, AMPA receptor, Development

## Abstract

NMDA receptors are heteromeric complexes that contribute to excitatory synaptic transmission and plasticity. The presence of specific variants of GluN2 subunits in these complexes enables diversity in NMDA receptor function and regulation. At brain synapses, there is a switch from slow GluN2B-mediated NMDA receptors to faster GluN2A-dominated NMDA receptors as well as an increase in the ratio of AMPA to NMDA receptors during early postnatal development. This glutamate receptor switch is observed across brain regions and is critical for synaptic maturation, circuit development, and associative learning. However, whether a similar receptor subunit switch occurs within pain processing neurons in the developing spinal cord remains untested. To investigate this, we performed whole-cell patch clamp recordings of excitatory synaptic responses from lamina II dorsal horn neurons of one to three week-old rats. We found that GluN2B and GluN2A both prominently contribute to NMDA receptor responses at neonatal lamina II synapses, with a small contribution from GluN2D as well. Surprisingly, we found that this molecular identity of NMDA receptor responses as well as the relative contribution of AMPA receptors versus NMDA receptors did not change at lamina II synapses across early postnatal development (P7 to P21). The lack of a developmental switch and persistence of slow-decaying GluN2B- and GluN2D-mediated synaptic responses throughout neuronal maturation in the dorsal horn has implications for understanding both the regulation of synaptic glutamatergic receptors as well as spinal mechanisms of pain processing.

## Introduction

The superficial layers of the spinal cord dorsal horn, laminae I and II, are a hub for processing pain-related sensory inputs [1]. Disruption in the balance of excitability within the superficial dorsal horn can drive chronic pain [2], and yet the specific molecular determinants that mediate synaptic signaling across dorsal horn neuron subpopulations remain poorly understood. The N-methyl-D-aspartate receptor (NMDAR) subtype of ionotropic glutamate receptors are critical mediators of synaptic transmission and plasticity throughout the nervous system. Within the superficial dorsal horn, synaptic NMDARs are implicated in both physiological and pathological mechanisms of pain processing [3]. Of the seven variants of genetically encoded NMDAR subunits (GluN1, GluN2A-D, and GluN3A-B), the majority of functional NMDAR heteromeric complexes contain two obligatory GluN1 subunits and two identical or different GluN2 subunits. The identity of the GluN2 subunit(s) shapes both the functional properties and regulation of synaptic NMDAR responses. For example, GluN2A-, GluN2B/C-, and GluN2D-containing diheteromeric NMDARs have fast (~ 50 ms), intermediate (~ 250 ms), and slow (> 1000 ms) deactivation rates, respectively [4]. While GluN2A-containing receptors dominate NMDAR responses at the majority of mature central synapses [4], synaptic NMDAR responses within the superficial dorsal horn can be mediated by GluN2A-, GluN2B- and/or GluN2D-containing NMDARs, depending on the specific synaptic input, laminae and developmental time point under study [5–7]. To gain a better understanding of the roles of individual NMDAR isoforms in dorsal horn nociceptive signalling, the biophysical and pharmacological properties of synaptic NMDARs need to be systematically studied and compared between distinct developmental stages for each subpopulation of superficial dorsal horn neuron.

In the first three weeks of postnatal brain development, there is a switch from predominant expression of GluN2B- and GluN2D- containing NMDARs at birth to a progressive increase in the relative expression of GluN2A-containing NMDARs [4]. The well-documented switch from GluN2B-mediated to GluN2A-mediated NMDAR responses at developing central synapses produces a dramatic decrease in NMDAR synaptic decay constants as well as an associated increase in the relative synaptic contribution of α-amino-3-hydroxy-5-methyl-4-isoxazolepropionate receptors (AMPARs) compared to NMDARs [8, 9]. The early dominance of slow deactivating GluN2B NMDARs promotes synaptic potentiation and maturation when there is correlated activity, while the subsequent switch to GluN2A dominance decreases synaptic strength and dampens further potentiation [10]. This synaptic switch is conserved across diverse brain regions and allows for maturation of associative learning abilities [11].

Our observation of GluN2B- and GluN2D-dominated NMDAR responses at a mature dorsal horn synapse [7] calls into question whether the canonical synaptic switch from GluN2B to GluN2A is conserved in the developing dorsal horn nociceptive network. Here, we use whole-cell patch clamp recordings in combination with subtype-specific GluN2 antagonists to systematically investigate excitatory glutamatergic responses and the contributions of GluN2 isoforms to the NMDAR component of responses at lamina II synapses across early postnatal spinal cord development.

## Results

### The biophysical properties of glutamatergic synaptic responses in lamina II neurons are conserved across early postnatal spinal cord development

**Fig. 1:**
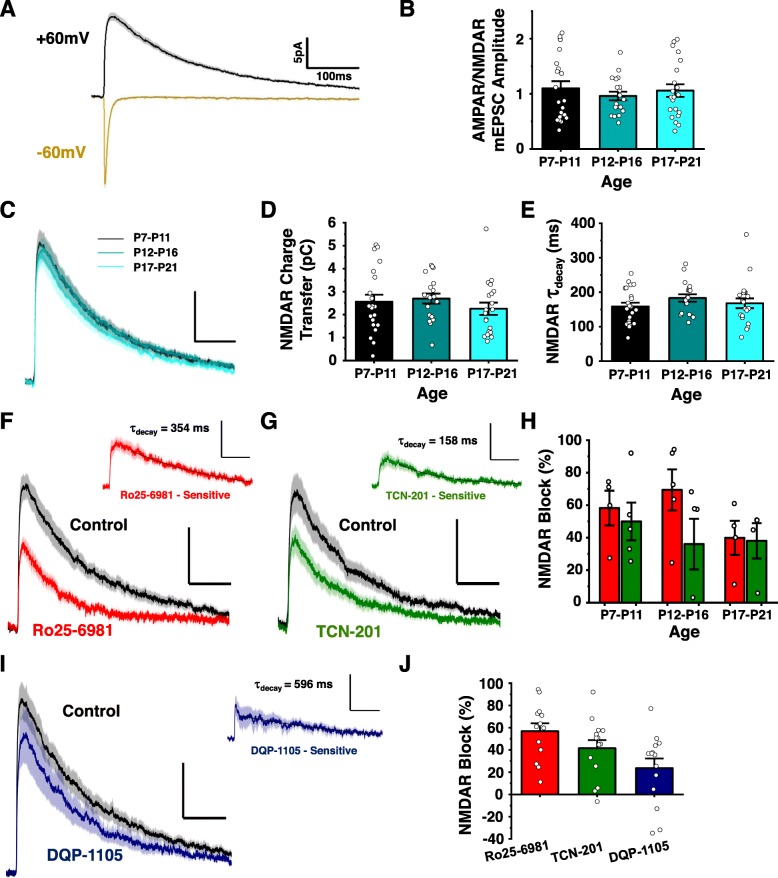
The biophysical and pharmacological properties of AMPAR- and NMDAR-mediated synaptic responses in lamina II neurons do not change across early postnatal development. (**A**) Averaged mEPSC traces of lamina II neurons (*n* = 62) at both − 60 mV (gold) and + 60 mV (black) from early postnatal (P7-P21) male rats. (**B**) Ratio of mEPSC peak amplitude at − 60 mV versus peak amplitude at + 60 mV (at 20 ms from onset) from lamina II neurons of postnatal rats aged P7 to P11 (black, *n* = 22 neurons), P12 to P16 (teal, *n* = 19 neurons), and P17 to P21 (aqua, *n* = 21 neurons) (**C**) Averaged mEPSC traces at + 60 mV for rats and neurons in the same postnatal age bins described in B. (**D**) Charge transfer through the NMDAR component of mEPSCs at + 60 mV (from 20 to 500 ms). (**E**) The exponential decay constants measured from mEPSCs at + 60 mV. (**F**) Averaged mEPSCs at + 60 mV before (black) and after (red) application of the GluN2B antagonist, 1 uM Ro25–6981 (*n* = 13). *Inset,* Ro25–6981-sensitive NMDAR mEPSC difference current. (**G**) Averaged mEPSCs at + 60 mV before (black) and after (green) application of the GluN2A antagonist, 10 uM TCN-201 (*n* = 14). *Inset*, TCN-201-sensitive mEPSC difference current. (**H**) The percent reduction in NMDAR charge transfer by both 1 uM Ro25–6981 (red, left bars) and 10 uM TCN-201 (green, right bars) were not significantly different across the various developmental time points: P7-P11 (*n* = 4,5), P12-P16 (*n* = 5,5), P17–21 (*n* = 4,4). (**I**) Averaged NMDAR mEPSCs before (black) and after (blue) application of 10 uM DQP-1105 (*n* = 14). *Inset*, 10 uM DQP-1105-sensitive NMDAR mEPSC difference current. (**J**) Percent NMDAR charge transfer reduction produced by administration of 1 uM Ro25–6981 (red, *n* = 13), 10 uM TCN-201 (green, *n* = 14) or 10 uM DQP-1105 (blue, *n* = 14). Cells with a negative blockade reflect an antagonist-independent run-up in NMDAR charge transfer [12] during the course of recording. All traces are presented as mean (darker line) +/− standard error (lighter shaded area). Scale bar x axes = 100 ms, y axes = 5 pA

To characterize postsynaptic glutamatergic responses at developing lamina II synapses, we performed whole-cell patch-clamp recordings on lamina II neurons from acute transverse spinal sections of postnatal day 7 (P7) to 21 (P21) male rats. We recorded miniature excitatory postsynaptic currents (mEPSCs) at − 60 mV and + 60 mV to study AMPAR- and NMDAR-mediated postsynaptic responses, respectively [7]. In lamina II neurons held at − 60 mV, we observed inward AMPAR-mediated mEPSCs with a peak amplitude of − 15.9 ± 0.9 pA and a decay constant (τ_decay_) of 11.1 ± 1.1 ms (*n* = 40 animals, 62 neurons, Fig. 1a). At a holding potential of + 60 mV, outward mEPSCs were much slower than mEPSCs at − 60 mV for the same neurons (Fig. 1a). To estimate the amplitude of the NMDAR component of mEPSCs at + 60 mV, we measured the mEPSC amplitude at 20 ms from onset, a timepoint of peak NMDAR response where the contribution from fast deactivating AMPARs is minimal [7]. The average amplitude of NMDAR mEPSCs at + 60 mV was 16.3 ± 0.7 pA, which was not significantly different from the absolute peak amplitude of mEPSCs at − 60 mV (*n* = 62 neurons, *p* = 0.68). The decay constant for NMDAR mEPSCs at + 60 mV (169 ± 7 ms) was significantly slower than that for AMPAR mEPSCs at − 60 mV (*n* = 62, *p* = 1.9e^− 31^, Fig. 1a). We next compared the biophysical properties of AMPAR and NMDAR mEPSCs at distinct time points in early postnatal development. Our developmental age bins of P7-P11, P12-P16, and P17-P21 correspond to postnatal periods that encompass the complete synaptic switch process across brain regions [11] and also correspond to periods of maximal change in primary afferent-induced postsynaptic responses and plasticity for developing dorsal horn neurons [13]. We found that the ratio of the peak amplitude of AMPAR mEPSCs at − 60 mV compared to the amplitude of NMDAR mEPSCs at + 60 mV (at 20 ms) remained unchanged (*p* = 0.67) near a value of 1 across the three postnatal time periods (P7-P11, 1.1 ± 0.1, *n* = 22 neurons; P12-P16, 1.0 ± 0.1, *n* = 19; P17-P21, 1.1 ± 0.1, *n* = 21; Fig. 1b). We therefore conclude that AMPARs and NMDARs contribute equally to excitatory synaptic responses in lamina II neurons across early postnatal rodent development.

To further investigate the NMDAR component of lamina II synaptic responses across early development (Fig. 1c), we calculated the charge transfer through mEPSCs at + 60 mV from 20 ms to 500 ms after event onset. Synaptic charge transfer during this time range is exclusively mediated by NMDAR and not AMPAR activity [7]. We found that there was no significant difference (*p* = 0.42) in the magnitude of NMDAR charge transfer between P7-P11 (*n* = 22), P12-P16 (*n* = 19), and P17-P21 (*n* = 21; Fig. 1d). Similarly, there was no significant change in decay constants for NMDAR mEPSCs at lamina II synapses across early postnatal development (Fig. 1c, e). Taken together, our results suggest that the relative contribution and biophysical properties of synaptic NMDARs do not change during the postnatal maturation of lamina II spinal cord neurons.

### Both GluN2B- and GluN2A-containing NMDARs mediate lamina II synaptic responses across early postnatal development

The lack of change in charge transfer and decay constants of NMDAR mEPSCs in developing lamina II neurons suggests that there may not be an early postnatal switch from GluN2B-containing NMDARs to GluN2A-containing NMDARs at these specific central synapses. In fact, a stable decay constant between approximately 160 ms and 180 ms across lamina II development (Fig. 1e) suggests that both GluN2A- and GluN2B-containing NMDARs may mediate synaptic responses throughout the maturation process [4]. We therefore used GluN2 subunit-specific pharmacological antagonists to directly explore the relative contribution of individual GluN2 subtypes to NMDAR-mediated mEPSCs. The contribution of GluN2B was investigated using the GluN2B-specific antagonist, Ro25–6981, at a concentration (1 μM) that inhibits recombinant GluN2B- but not GluN2A-containing diheteromeric NMDARs [14] and that we have shown to selectively inhibit GluN2B-like NMDAR responses at lamina I synapses [7] (see Methods for further discussions on NMDAR antagonists). NMDAR charge transfer was calculated for a 10-min baseline control recording period and compared to a time period where inhibition reached a steady-state level, typically after 40 minutes of drug administration. We found that administration of 1 μM Ro25–6981 resulted in a significant block of NMDAR mEPSCs at P7-P21 lamina II synapses (56.9 ± 7.1%, *n* = 13, *p* = 0.00035, Fig. 1f). To test for the contribution of GluN2A to NMDAR-mediated mEPSCs, we utilized the GluN2A-specific antagonist, TCN-201, at a concentration (10 μM) that selectively blocks only GluN2A-containing recombinant NMDARs [15] and that inhibits fast-decaying GluN2A-like but not slower decaying GluN2B-like synaptic NMDAR responses at rat lamina I synapses [7]. In contrast to mature lamina I neurons [7], we found that administration of 10 μM TCN-201 also robustly reduced NMDAR-mediated charge transfer at early postnatal lamina II synapses (41.6 ± 7.2%, *n* = 14, *p* = 0.00064, Fig. 1g). There was no significant difference between the baseline NMDAR charge transfers for 1 μM Ro25–6981- and 10 μM TCN-201-treated neurons (*p* = 0.90), suggesting that NMDAR biophysical properties did not diverge between lamina II neurons from the two treatment groups (Supplementary Fig. 1). To assess the GluN2 subunit-selectivity of the specific pharmacological antagonists, the drug-sensitive mEPSC difference current was graphed and fitted with an exponential decay constant (Fig. 1f, g). The 1 μM Ro25–6981-sensitive difference current displayed a decay constant of 354 ms (Fig. 1f), which falls within the deactivation range for GluN2B-containing diheteromeric NMDARs [4, 7]. The 10 μM TCN-201-sensitive difference current had a decay constant of 158 ms (Fig. 1g), consistent with the faster deactivation rate observed for GluN2A-containing NMDARs [4, 7]. From these findings, we conclude that synaptic NMDAR responses are primarily mediated by a combination of GluN2A- and GluN2B-containing receptors in P7 to P21 lamina II neurons.

We next explored whether the functional contribution of GluN2A versus GluN2B-containing receptors to synaptic responses changes during the postnatal maturation of lamina II neurons. To test this, we analyzed the effects of Ro25–6981 and TCN-201 on NMDAR charge transfer across the three distinct postnatal development periods: P7-P11(n_Ro_ = 4, n_TCN_ = 5), P12-P16 (n_Ro_ = 5, n_TCN_ = 5), and P17-P21 (n_Ro_ = 4, n_TCN_ = 4). Two-way ANOVA analysis revealed no significant difference in the magnitude of NMDAR block for either 1 μM Ro25–6891 (*p* = 0.24) or 10 μM TCN-201 (*p* = 0.72) between the three postnatal development periods (Fig. 1h). We therefore conclude that the relative contribution of GluN2A versus GluN2B to synaptic NMDAR responses in lamina II neurons does not change across early postnatal development.

### GluN2D has a minor contribution to mEPSCs at early postnatal lamina II synapses

Given that GluN2D-containing receptors prominently contribute to NMDAR responses at lamina I synapses [7], we next explored whether GluN2D NMDARs mediate a component of NMDAR mEPSCs at developing lamina II synapses. We utilized the GluN2D-selective antagonist, DQP-1105, at a concentration (10 μM) that inhibited approximately 85% of GluN2D NMDARs with negligible effects on GluN2A or GluN2B NMDARs [16] and that also inhibited native GluN2D-like synaptic NMDAR responses in lamina I neurons [7]. Treatment with 10 μM DQP-1105 resulted in a 23.7 ± 8.6% reduction in NMDAR charge transfer at P7-P21 lamina II synapses (*Control* 2.78 ± 0.32 pC, *DQP-1105* 2.07 ± 0.40 pC, *n* = 14, *p* = 0.033, Fig. 1i). The 10 μM DQP-1105-sensitive mEPSC difference current exhibited a decay constant of 596 ms (Fig. 4A), which is consistent with slow deactivating GluN2D-containing NMDARs [4, 7]. We found that the NMDAR blockade by DQP-1105 at lamina II synapses was less than that produced by Ro25–6981 or TCN-201 (Fig. 1j).

## Discussion

In this study, we used whole-cell patch-clamp recordings to show that spontaneous synaptic responses in lamina II neurons include a fast AMPAR-mediated component and a slower NMDAR-mediated component, which are equal in amplitude across early postnatal development. In contrast to mature lamina I neurons [7], we find that both GluN2A- and GluN2B-containing receptors prominently contribute to synaptic NMDAR responses in developing lamina II neurons, with a smaller contribution from GluN2D-containing receptors as well. Surprisingly, the roles of these three GluN2 NMDAR subunits in mediating lamina II synaptic responses remains constant across early postnatal development. We therefore conclude that unlike the vast majority of central synapses, there is no molecular switch in the composition of NMDARs and no change in the ratio between functional AMPARs and NMDARs at developing postnatal lamina II synapses.

Unlike in the brain [4], we found that the relative contribution of GluN2A- versus GluN2B-containing NMDARs to lamina II synaptic responses does not change between weeks one and three of postnatal development. This extends the initial pioneering work of Bardoni and colleagues demonstrating that the magnesium sensitivity and kinetics of synaptic NMDAR responses in laminae I and II neurons do not change in the first postnatal week of rat development [6]. The lack of the canonical downregulation of slow-decaying GluN2B- (and GluN2D-) mediated synaptic NMDARs for developing superficial dorsal horn neurons could potentially underlie the increased propensity for synaptic potentiation and central sensitization within these pain signaling circuits. Understanding the molecular differences in the developmental regulation of synaptic physiology and plasticity between the brain and spinal cord will therefore potentially inform the development of new therapeutic approaches for the treatment of chronic pain.

As NMDAR charge transfer and decay kinetics as well as blockade by specific GluN2 subunit antagonists varied considerably between individual lamina II neurons, the next step is to explore potential heterogeneity in the molecular composition of synaptic NMDARs between lamina II neuron subpopulations. The outer half of lamina II that we were recording from contains different types of excitatory and inhibitory nociceptive interneurons, which can be classified based on distinctions in morphology, excitability and neurochemistry [17], and, more recently, on RNA expression profiles [18, 19]. Future studies could combine biophysical and pharmacological characterization of synaptic NMDAR responses from individual lamina II neurons, as reported here, with neurochemical and morphological characterization of filled and labelled neurons. Moreover, single cell RNA sequencing from superficial dorsal horn tissue or even individual recorded neurons could be used to characterize the subtypes of NMDAR subunits that are functionally expressed within specific transcriptionally-identified subtypes of excitatory and inhibitory interneurons [18, 19]. This information is essential for uncovering the differential roles of excitatory glutamate receptor subtypes in synaptic plasticity and nociceptive function in subpopulations of dorsal horn neurons across development.

## Methods

### Animals

Male Sprague Dawley rats aged between postnatal day 7 to 21 (P7-P21) were ordered from Charles River and used for all experiments. All procedures and experimental protocols followed the guidelines set out by the Canadian Council on Animal Care regarding animal care and handling. Animal research protocols were authorized by the Animal Care Committee at Carleton University and the Animal Veterinary Services at The Heart Institute, where animals were housed.

### Spinal cord isolation and tissue sectioning

Early postnatal (P7-P21) male Sprague Dawley rats were anaesthetized using intraperitoneal injections of 20% (wt/vol) urethane (Sigma-Aldrich) at 0.1 mL/10 g of weight. The spinal cord was excised immediately following anesthesia and placed in an ice-cold, bubbled (5% CO_2_, 95% O_2_) sucrose cutting solution containing (in mM): 50 sucrose, 92 NaCl, 15 D-(+)-glucose, 26 NaCO_3_, 5 KCl, 1.25 NaH_2_PO_4_, 0.5 CaCl_2_, 7 MgSO_4_, and 1 mM kynurenic acid. The trimmed spinal segment containing L3-L6 was mounted on its ventral surface to a piece of agar, which was then glued vertically to a vibratome sectioning chuck. Transverse (400 μm) slices were generated using a Leica VT1200S vibratome. Spinal cord tissue sections were placed into a bubbled sucrose cutting solution that did not contain kynurenic acid and was heated to ~ 33.9 °C using a water bath (Fisher-Scientific). The slices were allowed to recover for 40 min at this temperature before the solution chamber was removed from the water bath and allowed to passively reach room temperature (~ 23 °C).

### Whole-cell patch-clamp electrophysiological recordings on lamina II spinal cord neurons

Spinal cord sections were visualized under a Zeiss microscope and acutely perfused with artificial cerebral spinal fluid (aCSF) containing (in mM): 125 NaCl, 3 KCl, 26 NaCO_3_, 1.25 NaH_2_PO_4_, 2 CaCl_2_, 1 MgCl_2_, and 20 D-(+)-glucose, 10 μM bicuculline (Tocris Bioscience), 10 μM strychnine, 10 μM Cd^2+^, and 0.5 μM TTX (Alomone Labs). Neurons located in the outer half of lamina II were visualized first under 10x based on their location in the substantia gelatinosa, followed by 40x magnification. Patch-clamp recording borosilicate glass pipettes of 5 to 11 M Ω resistance were pulled using a Sutter Flaming micropipette puller and fire-polished. Pipettes were filled with an internal solution containing (in mM): 105 D-gluconic acid, 105 CsOH, 17.5 CsCl, 10 HEPES, 10 EGTA, 2 Mg-ATP, 0.5 Na2-GTP (pH = 7.25; 295 mOsm). Voltage-clamp recordings were obtained from whole-cell patch-clamped neurons at room temperature using a Multi-clamp 700B amplifier (Molecular Devices, Sunnyvale, CA, USA), Digidata 1550 Data Acquisition System (Molecular Devices) and a personal computer running pClamp10.7 software. Initial recordings were obtained at − 60 mV. Following 120 s of recording at − 60 mV, the voltage was incrementally adjusted to + 60 mV to remove blockade of NMDAR responses by magnesium ions. Pharmacological blockers were added following acquisition of a minimum of 20 mEPSC events at + 60 mV in a 10-min baseline recording period.

### Pharmacological inhibition of specific NMDAR GluN2 subunits

Unless otherwise stated, all chemicals used in this experiment were obtained from Sigma-Aldrich. For GluN2 subtype-specific antagonists, 1 μM Ro-25-6981 (Tocris Bioscience) was acutely perfused to selectively inhibit GluN2B-containing NMDARs [14]. It should be noted that NMDAR inhibition by Ro25–6981 is activity-dependent [14], which means that NMDAR mEPSC blockade by acute perfusion of 1 μM Ro25–6981, as performed here, may underrepresent the fraction of synaptic current that is mediated by GluN2B NMDARs. We applied TCN-201 (Tocris Bioscience) at a concentration (10 μM) that robustly blocks GluN2A diheteromeric NMDARs but not GluN2B, GluN2C, or GluN2D diheteromeric NMDARs [15]. The potency of GluN2A NMDAR blockade by TCN-201 decreases with increasing glycine concentration [15], but with basal glycine concentrations in the rat spinal cord at approximately 13 μM [20], application of 10 μM TCN-201 will still block approximately 90% of GluN2A diheteromeric NMDARs. As 10 μM TCN-201 has recently been reported to also block over 60% of triheteromeric NMDARs containing both GluN2A and GluN2B [21], it is possible that some of the TCN-201-sensitive NMDAR current is also mediated by GluN2A/2B triheteromeric NMDARs. The GluN2D (and GluN2C) subunit-specific inhibitor, 10 μM DQP-1105 (Tocris Bioscience) [16], was used to assess the contribution of GluN2D-containing NMDARs to synaptic responses in early postnatal lamina II neurons. Each antagonist was dissolved from stock concentrations in DMSO (10 mM) to the final concentrations listed above into bubbled aCSF, without any visible precipitate forming. Antagonists were perfused during recording for an average of 38.2 ± 1.7 min (*n* = 41).

### Statistical analysis

All results are presented as average ± standard error of the mean (SEM). Statistical comparisons of data were performed using the following (where applicable): Student’s paired t-test, student’s unpaired t-test (unequal variance), and one- or two- way ANOVA followed by a Tukey’s test for means comparisons. Results were interpreted as statistically significant at *p* < 0.05.

## Supplementary information

**Additional file 1 **Supplementary Fig. 1 Both GluN2B and GluN2A contribute prominently to NMDAR responses at lamina II synapses. Average NMDAR charge transfer following application of 1 uM Ro25–6981 (red, *n* = 13) or 10 uM TCN-201 (green, *n* = 14), with the baseline NMDAR charge transfer for the corresponding cells shown in the black bars to the left. Note the lack of difference between baseline charge transfer values for Ro25–6981- versus TCN-201-treated cells.

## Data Availability

All data generated or analysed during this study are included in this published article (and its supplementary information files). Any additional information related to the current study is available from the corresponding author on reasonable request.

## Abbreviations

AMPAR: α-amino-3-hydroxy-5-methyl-4-isoxazolepropionate receptor
mEPSC: miniature excitatory postsynaptic current
NMDAR: N-methyl-D-aspartate receptor
Postnatal day 7: P7
