# Supplementary material for: Conserved contributions of NMDA receptor subtypes to synaptic responses in lamina II spinal neurons across early postnatal development
Source: Mol Brain. 2020 Mar 5;13:31. doi: 10.1186/s13041-020-00566-9 (PMC7057509; doi:10.1186/s13041-020-00566-9)
Supplement: Supplementary file 1 — Additional file 1 Supplementary Fig. 1 Both GluN2B and GluN2A contribute prominently to NMDAR responses at lamina II synapses. Average NMDAR charge transfer following application of 1 uM Ro25–6981 (red, n = 13) or 10 uM TCN-201 (green, n = 14), with the baseline NMDAR charge transfer for the corresponding cells shown in the black bars to the left. Note the lack of difference between baseline charge transfer values for Ro25–6981- versus TCN-201-treated cells. [file 13041_2020_566_MOESM1_ESM.pptx]

## Slide 1
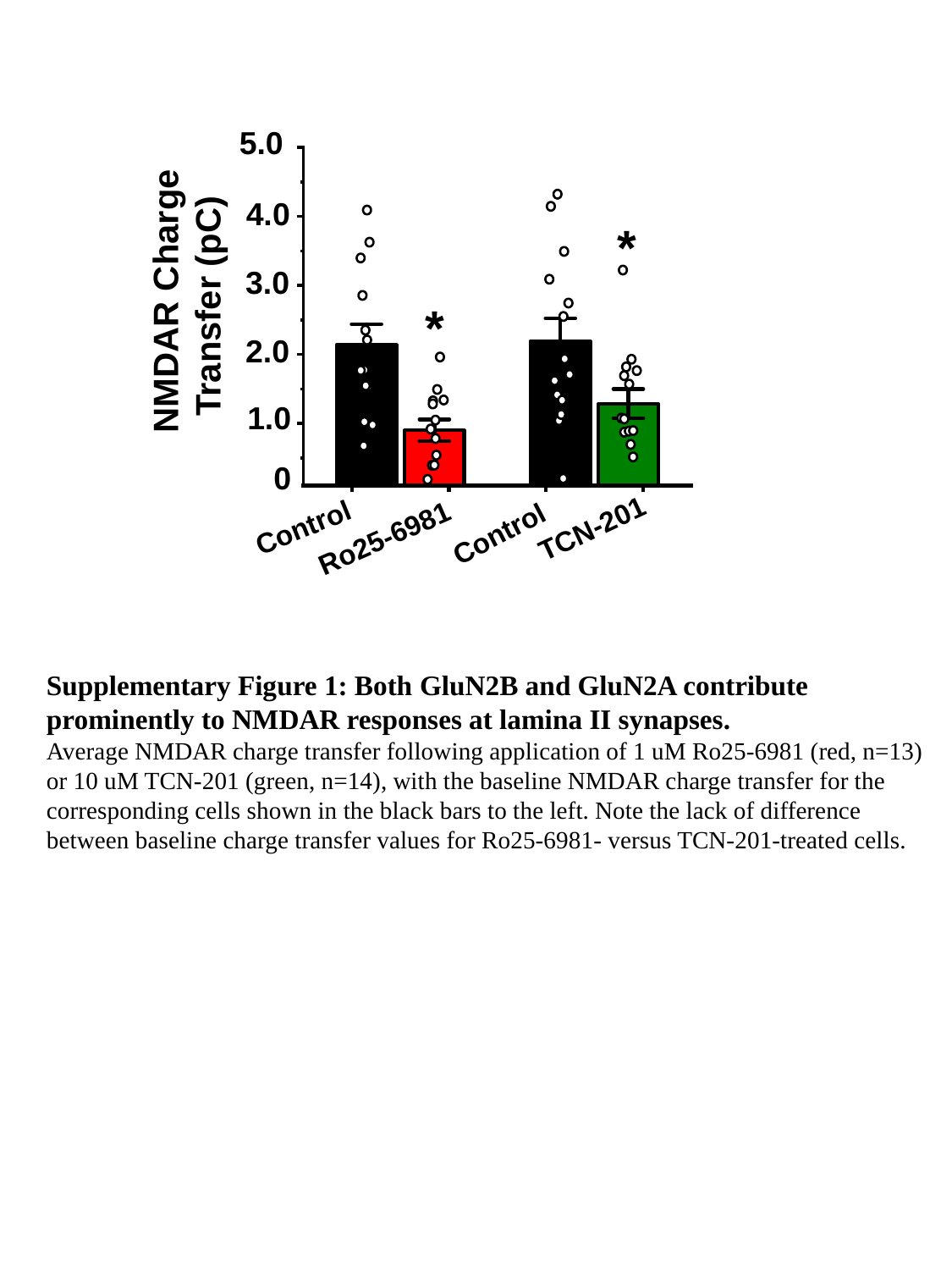

5.0
3.0
 NMDAR Charge Transfer (pC)
2.0
1.0
0
TCN-201
Control
Control
Ro25-6981
4.0
*
*
Supplementary Figure 1: Both GluN2B and GluN2A contribute prominently to NMDAR responses at lamina II synapses.
Average NMDAR charge transfer following application of 1 uM Ro25-6981 (red, n=13) or 10 uM TCN-201 (green, n=14), with the baseline NMDAR charge transfer for the corresponding cells shown in the black bars to the left. Note the lack of difference between baseline charge transfer values for Ro25-6981- versus TCN-201-treated cells.
